# Respirometric analysis of *Penicillium simplicissimum *growth in solid-state fermentation using *Jatropha *cake as culture medium

**DOI:** 10.1186/1753-6561-8-S4-P220

**Published:** 2014-10-01

**Authors:** Mateus Godoy, Sevastianos Roussos, Denise Freire

**Affiliations:** 1 Universidade Federal do Rio de Janeiro, Laboratório de Biotecnologia Microbiana, Departamento de Bioquímica, Instituto de Química, 21941-909, Rio de Janeiro, RJ, Brazil; 2 Aix-Marseille Université (AMU), Institut Mediterranéen de Biodiversité et d'Ecologie Marine et continentale (IMBE), Équipe de Biotechnologies et Bioremédiation (B&B), 13397, Marseille, France

## 

Jatropha cake (JC), a toxic residue derived from the extraction of the oil from *Jatropha curcas *seeds, can be used as a culture medium for solid-state fermentation (SSF). Through this process, it is possible to detoxify and add value to this solid residue through the production of enzymes of biotechnological interest. However, one of the major technological bottlenecks in SSF is the microbial growth control. This study aims to monitor the growth of the fungus *P. simplicissimum *in JC by respirometry and compare this process of lipase production in tray-type bioreactor with the one in Raimbault columns.

First, the experiments were carried out and optimized in tray-type bioreactors. After two sequential experimental designs - Plackett-Burman and Central Composite Rotatable Design - a production of 140U/g was reached, representing a 9-fold increase of the initially obtained activity.

Based on the conditions indicated by the experimental design, the fermentations were carried out on Raimbault columns [1]. In these experiments, it was possible to follow the profile of CO_2 _production by *P. simplicissimum *through respirometric analysis. It was observed a high and fast production of CO_2 _over the first 24h of growth. After this time, there is a reduction of the CO_2 _release and the fungus achieve its basal metabolism around 96h. In spite of the intense fungal growth, a low lipase production was reached (50U/g), in contrast with the one obtained in tray-type bioreactors. This behavior is probably due to the strong compression of the solid culture medium and, consequently, the difficulty of heat and mass transfer, even under forced aeration. In order to reduce the compaction of the medium, a mixture of JC with sugarcane bagasse was used. Sugarcane bagasse (SCB) is widely used in SSF with other agroindustrial residue for better "structure" the culture medium, avoiding compaction problems [2]. After some assays, a medium composed of 15% (m/m) of SCB and 85% (m/m) of JC was used. After sugarcane bagasse addition, the lipase production was 160U/g, near to the one obtained in tray-type bioreactor.

The production profile of CO_2 _is similar to those obtained by other fungi cultivated in SSF and in other culture mediums [3]. However, in both curves obtained, an unusual small change in the stage of metabolism in the first 24h was observed in all experiments, probably due to the waste toxicity. The phorbol ester content (the major toxic component) was 70% reduced reaching a final concentration of 676 μg/g.

From these experiments, it is observed that (i) for SSF in columns bioreactors with JC, the use of sugarcane bagasse (or other inert raw material) to structure the culture medium is essential, avoiding compaction, (ii) the fungus *P. simplicissimum *reached a high lipase production when grown in Raimbault columns with JC and SCB, reaching a production of 160U/g, (iii) through the respirometric system to monitoring the fungal growth, it is possible to see the stages of metabolism change of the fungus, allowing further studies and (iv) the fungus was able to reduce 70% of the phorbol esters content after 26h of fermentation.
